# Pancreatic stone protein/regenerating protein is a potential biomarker for endoplasmic reticulum stress in beta cells

**DOI:** 10.1038/s41598-019-41604-4

**Published:** 2019-03-26

**Authors:** Stephen Stone, Damien Abreu, Jana Mahadevan, Rie Asada, Kelly Kries, Rolf Graf, Bess A. Marshall, Tamara Hershey, Fumihiko Urano

**Affiliations:** 1 Department of Pediatrics, Washington University School of Medicine, St. Louis, Missouri United States of America; 2 Department of Medicine, Division of Endocrinology, Metabolism, and Lipid Research, Washington University School of Medicine, St. Louis, Missouri United States of America; 3 Division of Biology & Biomedical Sciences, Washington University School of Medicine, St. Louis, Missouri United States of America; 4 Department of Visceral & Transplantation Surgery, University Hospital Zurich, Zurich, Switzerland; 5 Department of Cell Biology and Physiology, Washington University School of Medicine, St. Louis, Missouri United States of America; 6 Departments of Psychiatry, Neurology, and Radiology, Washington University School of Medicine, St. Louis, Missouri United States of America; 7 Deparment of Pathology and Immunology, Washington University School of Medicine, St. Louis, Missouri United States of America; 8 Department of Biochemistry, Institute of Biomedical & Health Science, Hiroshima University, Hiroshima, 734-8553 Japan; 9 Present Address: MilliporeSigma (SAFC), St. Louis, Missouri United States of America

## Abstract

Endoplasmic reticulum (ER) stress in beta cells is an important pathogenic component of both type 1 and type 2 diabetes mellitus, as well as genetic forms of diabetes, especially Wolfram syndrome. However, there are currently no convenient ways to assess ER stress in beta cells, raising the need for circulating ER stress markers indicative of beta cell health. Here we show that pancreatic stone protein/regenerating protein (PSP/reg) is a potential biomarker for ER stressed beta cells. PSP/reg levels are elevated in cell culture and mouse models of Wolfram syndrome, a prototype of ER stress-induced diabetes. Moreover, PSP/reg expression is induced by the canonical chemical inducers of ER stress, tunicamycin and thapsigargin. Circulating PSP/reg levels are also increased in some patients with Wolfram syndrome. Our results therefore reveal PSP/reg as a potential biomarker for beta cells under chronic ER stress, as is the case in Wolfram syndrome.

## Introduction

Diabetes mellitus is a global epidemic, affecting an estimated 30.3 million people in the United States^1^. It causes heavy financial burdens at both the personal and the public health level due to the longitudinal medical care and self-management education required to properly control this disease^2^. Regardless of its etiology, diabetes is characterized by an absolute or relative deficiency in insulin production by pancreatic beta cells. As the major site of insulin biosynthesis, the endoplasmic reticulum (ER) is particularly important for beta cell function. The ER is responsible for proper protein folding and sorting as well as calcium signaling and storage. Perturbations to ER homeostasis have direct implications for determining between cell life and death^3,4^. Accordingly, ER dysfunction, or ER stress, is directly involved in the beta cell pathogenesis of both type 1 (T1DM) and type 2 diabetes (T2DM)^5–9^. In both forms of diabetes, a combination of genetic and metabolic insults to ER homeostasis result in a complex cellular response that drives calcium efflux from the ER and activates the unfolded protein response^4^. Depending on the severity and duration of the stress, these responses by the ER can culminate in beta cell death^4,10,11^.

Wolfram syndrome (OMIM 222300) is considered a prototype of human ER stress disease^12^. As a monogenic, neurodegenerative form of diabetes, stemming from ER dysfunction, Wolfram syndrome is a prime model for studying the pathophysiology of ER stress in beta cells. Most cases of this rare autosomal recessive disorder are caused by mutations in the *WFS1* gene, which encodes an ER transmembrane protein^13^. While the function of this protein is still not clear, accumulating evidence suggests that disease-causing alleles promote chronic, unresolvable ER stress in neural and endocrine tissues. This leads to cellular dysfunction and ultimately cell death, which typically first manifests as juvenile-onset diabetes mellitus, followed by bilateral optic nerve atrophy^14^. Animal and cell models of Wolfram syndrome are increasingly recapitulating the aspects of ER stress-induced beta cell pathology that lead to disease. More specifically, upregulation of ER stress markers, reduced beta cell mass, and defects in glucose-stimulated insulin secretion are observed in whole body and beta cell-specific WFS1 knockout mice, as well as rodent beta cell models of WFS1 depletion^15,16^. It is therefore clear that by leveraging our understanding of Wolfram syndrome as a monogenic disorder of ER stress, we can identify novel biomarkers and molecular pathways pertinent to more common diseases resulting from ER dysfunction. Such biomarkers will be very useful as researchers pursue clinical trials for Wolfram syndrome and other metabolic disorders in which beta cell ER stress is an integral component.

This study aimed to identify differentially expressed proteins in rodent models of Wolfram syndrome that could serve as biomarkers of ER stress in beta cells. It then evaluated the potential of one of the candidate proteins, pancreatic stone protein/regenerating protein (PSP/reg), as a clinical biomarker in subjects with Wolfram syndrome. There are several genes in the PSP/reg family, and PSP/reg has various alternative names including: regenerating protein 2, lithostathine-2, pancreatic thread protein, and protein-X^17^. These studies examine the PSP/reg1 family, where there is closest homology between mouse *Reg2* and rat *Reg1*^18^.

## Results

### Loss of *Wfs1* leads to induction of PSP/reg

Beta cells respond to ER stress through the activation of transcriptional and translational programs aimed at resolving the stress^19^. We hypothesized that beta cells in Wolfram syndrome would activate signaling pathways that could be utilized as clinical biomarkers of beta cell ER stress. In order to test this hypothesis, we measured differentially expressed proteins in a mouse model of Wolfram syndrome, a genetic model of chronic beta cell ER stress. Two-dimensional gel electrophoresis was used to resolve the proteomes of islets derived from two 17-week-old *Wfs1* beta cell-specific male knockout mice and two age-matched littermate control male mice. Due to the relatively small amount of protein that can be isolated from the mouse islets, we chose to combine islets from 2 mice in order to obtain enough protein to peform proteomics via mass spectroscopy. Of the approximately 450 spots analyzed in the molecular mass range of 5–110 kDa, 72 protein spots showed a difference of 1.5-fold or greater between *Wfs1* knockout islets and control islets. As only 2 mice were used in each condition, we were unable to calculate statistical significance on these results. To refine our search for potential biomarkers of ER stress, the top 11 most upregulated spots in *Wfs1* knockout islets were subjected to mass spectroscopy, resulting in the identification of 7 unique proteins (Supplementary Fig. 1a and Supplementary Table 1). Several of the peptide fragments were predicted to be the same protein, likely representing different post-translational modification states of those proteins.

Notably, many of the proteins identified are digestive enzymes (Supplementary Table 1). This includes chymotrypsinogen B, trypsin 4 precursor, trypsinogen 7 precursor, and chymotrypsin-like elastase. Pancreatic alpha-amylase plays a role in carbohydrate metabolism. In keeping with our hypothesis, we found protein disulfide isomerase to be 3.72-fold higher in *Wfs1* knockout islets compared to control islets. Protein disulfide isomerase is an ER resident protein involved in protein folding by catalyzing the formation of disulfide bonds^20^. The upregulation of protein disulfide isomerase in *Wfs1* beta cell-specific knockout islets suggests that ER stress leads to adaptive changes that promote proper protein folding via the formation of disulfide bonds^21^.

Pancreatic stone protein (PSP/reg) was the most upregulated protein in *WFS1* knockout islets identified by our analysis. Two isoforms of PSP/reg were detected: a higher molecular weight isoform, which was upregulated 3.98-fold compared to wild-type (WT) islets, and a lower molecular weight isoform, which was upregulated 3.55-fold (Supplementary Fig. 1b). This was of particular interest to us because PSP/reg is a small secreted peptide (approximately 16 kDa) that has been studied primarily for its role in islet regeneration, suggesting that PSP/reg may have properties that promote beta cell health and adaptation to ER stress^17,22^.

Given that PSP/reg was elevated in islets isolated from beta cell-specific *Wfs1* knockout mice, we hypothesized that PSP/reg would co-localize with beta cells in the islet. To test this hypothesis, we performed immunohistochemistry on pancreatic sections obtained from 3 whole body Wfs1-knockout mice and 3 wild-type control mice. As demonstrated in previous publications, PSP/reg is strongly expressed in acinar tissue^23^. Insulin and PSP staining co-localized within both *Wfs1*+/+ and *Wfs1*−/− islets. Some of the *Wfs*1−/− islets demonstrated a subtle increase in PSP/reg staining (Supplementary Fig. 1c).

**Figure 1:**
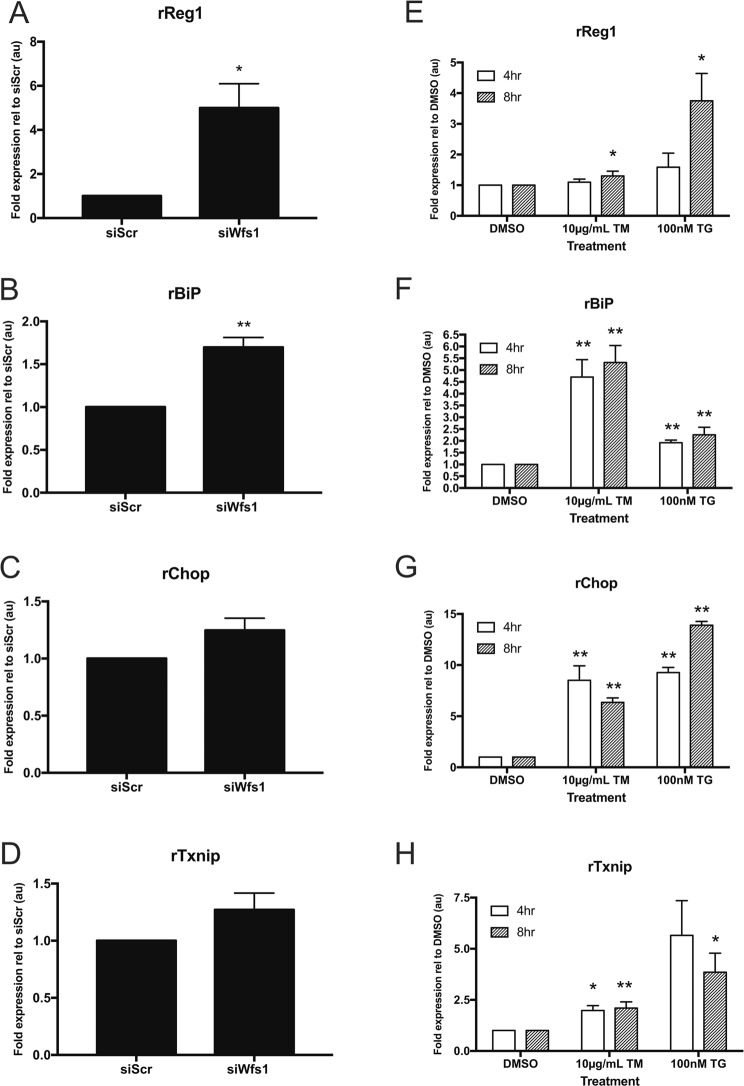
PSP/reg is induced by endoplasmic reticulum stress. (**A**–**D**) INS-1 832/13 cells were transfected with siRNA directed against *WFS1* (siWFS1) or control scrambled siRNA (siScr). Quantitative real-time PCR was used to measure gene expression of (**A**) *Reg1*, (**B**) *Bip* (**C**) *Chop*, and (**D**) *Txnip*. Knockdown of *WFS1* increased *Reg1* expression 5-fold. *Bip* expression was increased by ~70%, *Chop* expression by ~25%, and *Txnip* expression by ~25%, relative to control. (**E**–**H**) INS-1 832/13 cells were treated with two chemical inducers of ER stress, tunicamycin (TM) and thapsigargin (TG), at the doses specified. DMSO was used as a vehicle control. Quantitative real-time PCR was used to measure gene expression of (**E**) *Reg1*, (**F**) *Bip* (**G**) *Chop*, and (**H**) *Txnip*. The expression of *Reg1* was significantly increased by TM and TG after 8 hours of treatment. As expected, TM and TG treatment led to upregulation of *Bip*, *Chop* and *Txnip*. *p < 0.05, **p < 0.01. Statistical significance was determined by an unpaired two-tailed t-test between a treated condition and its corresponding control condition.

Given our proteomic data showing that PSP/reg protein levels increase in *Wfs1* knockout islets, we hypothesized that *Wfs1* depletion would also increase *PSP*/*reg* expression at the transcriptional level. To test this hypothesis, we monitored gene expression of *PSP*/*reg* in a rat insulinoma (INS-1 832/13) cell line expressing small interfering RNA (siRNA) directed against *Wfs1*. Consistent with our islet data, cells depleted of *Wfs1* expressed a 5-fold increase in *Reg1* compared to the control cells (Fig. 1A). This suggests that PSP/reg expression may be negatively regulated by WFS1.

### PSP/reg is induced by ER stress

*WFS1* has been shown to negatively regulate ER stress^24,25^. We therefore anticipated that loss of *Wfs1* expression would lead to induction of canonical ER stress marker genes such as *Bip*, *Chop* and *Txnip*. To test this hypothesis, we measured expression levels of these ER stress markers by quantitative real-time PCR (qRT-PCR) using siRNA directed against *Wfs1* in INS-1 832/13 cells. As expected, *BiP* expression was increased in *Wf*s1-knockdown cells compared to control cells (Fig. 1B). Expression of *Chop* and *Txnip* was also mildly induced by *Wfs1*-knockdown, although it did not reach statistical significance (Fig. 1C and D). *BiP* is an ER stress-inducible molecular chaperone whose upregulation indicates activation of signaling pathways to restore cellular homeostasis^26^. Conversely, *Chop* and *Txnip* are ER stress-inducible molecules whose chronic activation leads to apoptosis^27,28^. Taken together, our findings suggest that transient knockdown of *Wfs1* stimulates a baseline induction of ER stress in beta cells. These results are consistent with previous reports of *Wfs1* loss-of-function models in which beta cells exhibit heightened ER stress and thus increased susceptibility to ER stress-mediated cell death^12,16^.

Since levels of PSP/reg expression were elevated in animal and cell models of Wolfram syndrome, and Wolfram syndrome is itself a model of ER stress disease, we hypothesized that PSP/reg would also be broadly inducible by ER stress. If true, other known endoplasmic reticulum stressors would increase the expression of *PSP*/*reg* genes. To test this hypothesis, we treated INS-1 832/13 cells with tunicamycin (TM) and thapsigargin (TG), two well-known chemical inducers of ER stress. TM causes ER stress by inhibiting N-linked glycosylation in the ER, thus disrupting protein folding^29^. TG elicits ER stress by irreversibly inhibiting the sarcoplasmic/endoplasmic reticulum calcium ATPase (SERCA), thus disrupting calcium homeostasis through ER calcium depletion^29,30^. To assess the dynamics of PSP/reg induction by ER stress, we performed a time course experiment in which we treated INS-1 832/13 cells with TM (10 µg/mL), TG (100 nM), or DMSO (control). We found that both TM and TG lead to significant upregulation of *Reg1* over the span of 8 hours, but not over 4 hours. This suggests that the induction of *Reg1* may be part of a late phase response to ER stress. Additionally, TG elicited a much stronger induction of *Reg1* expression compared to TM, suggesting that *Reg1* may be more sensitive to changes in calcium homeostasis than disruptions in protein folding (Fig. 1E). As expected, both TM and TG treatment increased the expression of the canonical ER stress genes *Bip*, *Chop* and *Txnip*. Notably, the induction of these genes occurred within 4 hours, further indicating that ER stress-induced *Reg1* expression is slow in comparison to other canonical, early ER stress response genes (Fig. 1F–H).

### PSP/reg levels in human subjects with Wolfram syndrome

**Figure 2:**
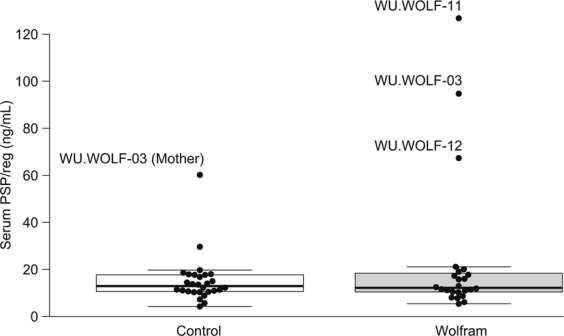
Serum Levels of PSP/reg in Human subjects with Wolfram Syndrome. PSP/reg levels were obtained from all available subjects attending the 2014 Wolfram syndrome research clinic. This included 28 subjects with Wolfram syndrome, and 28 control subjects (parents or siblings of subjects with Wolfram syndrome). The mean PSP/reg level in the Wolfram group was 23.1 ng/mL (SD 29.9), compared to 15.1 ng/mL (SD 10.2) in the control group. The median PSP/reg level in the Wolfram group was 12.2 ng/mL (interquartile range 10.4–18.1 ng/mL), compared to 12.9 ng/mL (interquartile range 10.6–17.7 ng/mL). Despite the similarities between the Wolfram and control groups, we noted that 3 subjects with Wolfram syndrome had relatively elevated levels of PSP/reg (WU-WOLF-03, WU-WOLF-11, WU-WOLF-12). From the control group one subject (WU-WOLF-03 (Mother)), also had relatively elevated levels of PSP/reg.

Since the expression of PSP/reg was elevated in mouse and cell models of Wolfram syndrome, we were interested in determining whether PSP/reg might be elevated in patients with Wolfram syndrome. Accordingly, we measured circulating PSP/reg levels in the serum of all of the available subjects attending the 2014 Wolfram syndrome research clinic. This included 28 subjects with Wolfram syndrome, and 28 control subjects (parents or siblings of subjects with Wolfram syndrome). The mean PSP/reg level in the Wolfram syndrome group was 23.1 ng/mL (SD 29.9), compared to 15.1 ng/mL (SD 10.2) in the control group. The median PSP/reg level in the Wolfram syndrome group was 12.2 ng/mL (interquartile range 10.4–18.1 ng/mL), compared to 12.9 ng/mL (interquartile range 10.6–17.7 ng/mL) in the control group. (Fig. 2)

Despite the similarities between both groups, we noted that 3 subjects with Wolfram syndrome had relatively elevated levels of PSP/reg. We carefully evaluated the medical history of these 3 subjects for age, gender, specific WFS1 gene mutations, and the age of onset for the major clinical components of Wolfram syndrome. This included diabetes mellitus (DM), optic atrophy (OA), hearing loss, and diabetes insipidus (DI). We compared the PSP/reg level to their fasting glucose and c-peptide levels (Table 1).

**Table 1:** Clinical Data Regarding 3 Subjects with Wolfram Syndrome with the Highest Levels of PSP/reg.

Subject	Age	Gender	PSP/reg (ng/mL)	Fasting		WFS1		Age of disease onset			
				Glucose (mg/dL)	C-peptide (ng/mL)	Allele 1	Allele 2	DM	OA	Hearing loss	DI
WU.WOLF-03	22	M	94.7	279	0.65	c.1230_1233delCTCT; p.V412fs*440	c.1243_1245delGTC; pV415del	4	6	6	6
WU.WOLF-11	12	M	126.8	278	0.4	c.376G>A; p.A126T	c.1838G>A; p.W613*	7	7	8	8
WU.WOLF-12	26	M	67.3	195	0.14	c.320G>A; p.G107E	c.1882C>T; p.R629W	6	7	7	15

Subject number, age, gender, and genotype of these subjects. Age (years) of clinical onset of DM (diabetes mellitus), OA (optic atrophy), hearing loss, DI (diabetes insipidus). Also listed is the subjects fasting PSP/reg, glucose, and C-peptide levels.

Subject WU.WOLF-03 is male subject who was 22 years old at the time of the study. He carries two deletions in the *WFS1* gene. The first *WFS1* allele is a 4 base pair deletion, resulting in a frameshift and a premature stop codon, resulting in a truncated protein (c.1230_1233_CTCT; p.V412fs*440_∗). The second *WFS1* allele is a 3-base pair deletion. This mutation eliminates a valine at position 415 (c.1243_1245delGTC; p.V415del). WU.WOLF-03 was diagnosed with diabetes mellitus at age 5. He was diagnosed with optic atrophy, hearing loss, and diabetes insipidus at age 6. He had a relatively elevated PSP/reg level, 94.7 ng/mL. His fasting glucose was 279 mg/dL, and fasting c-peptide was 0.65 ng/mL. This subject’s mother, WU.WOLF-03 (mother), was also an outlier amongst the control group. Her PSP/reg level was 60.2 (ng/mL). It is possible that the maternally inherited allele may be particularly deleterious and effective at inducing PSP/reg.

Subject WU.WOLF-11 is a male subject who was 12 years old at the time of the study. He carries a missense mutation and a nonsense mutation of the *WFS1* gene. The first *WFS1* allele carries a G to A mutation, resulting in an alanine to threonine mutation at position 126 (c.376G>A; p.A126T). The second *WFS1* allele carries a G to A mutation, resulting in a premature stop codon at position 613 (c.1838G>A; p.W613X). WU.WOLF-11 was diagnosed with diabetes mellitus and optic atrophy at age 7. He was diagnosed with diabetes insipidus at age 8, and hearing loss at age 9. WU.WOLF-11 had the highest measured PSP/reg level, 126.8 ng/mL. His fasting glucose was 278 mg/dL, and fasting c-peptide was 0.65 ng/mL.

Subject WU.WOLF-12 is a male subject who was 26 years old at the time of the study. He carries two missense mutations in the *WFS1* gene. The first *WFS1* allele carries a G to A mutation, this results in a glycine to glutamine mutation at position 107 (c.320G>A; p.G107E). The second *WFS1* allele carries a C to T mutation, resulting in an arginine to tryptophan mutation at position 629 (c.1882C>T; p.R629W). His PSP/reg level was elevated at 67.3 ng/mL. His fasting glucose was 195 mg/dL, and his fasting c-peptide was 0.14 ng/mL. This subject underwent a mixed-meal tolerance test. At 30 minutes, his glucose was 207 mg/dL, and his c-peptide was 0.3 ng/mL.

We hypothesized that serum PSP/reg levels may be positively correlated to disease severity in Wolfram syndrome. Therefore, we compared serum PSP/reg levels to fasting C-peptide levels obtained from the 28 subjects participating in the 2014 Wolfram syndrome clinic. We noted that the 3 subjects with relatively high serum PSP/reg all had lower levels of fasting C-peptide (0.14–0.65 ng/mL). However, there were several subjects within the same range of fasting C-peptide who had normal PSP/reg levels (Supplementary Fig. 2a). We also compared serum PSP/reg levels to the age of onset for the major clinical components of Wolfram syndrome, including diabetes mellitus, optic nerve atrophy, hearing loss, and diabetes insipidus. We did not identify any clear correlation between the age of onset of these conditions and serum PSP/reg levels (Supplementary Fig. 2b–e). We also compared the serum PSP/reg levels to the Wolfram Unified Rating Scale (WURS)^31^ obtained from these subjects during the 2014 Wolfram syndrome clinic. We did not identify any clear correlation between serum PSP/reg levels and the total, physical, or behavioral WURS scores (Supplementary Fig. 2f–h). Anecdotally, however the clinical providers at the Wolfram syndrome clinic felt that the subjects with the highest PSP/reg were some of the more severely affected subjects in the cohort.

## Discussion

**Figure 3:**
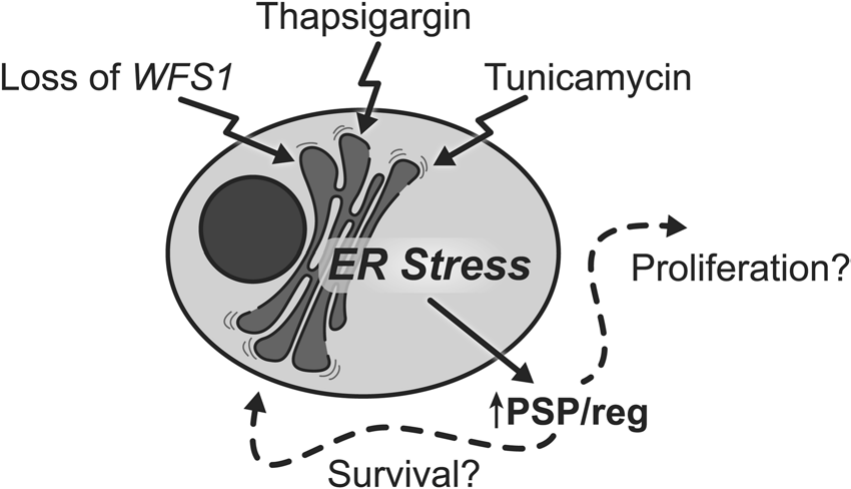
Proposed Model of PSP/reg in the ER stress response. ER stress either via genetic (loss of *WFS1*) or environmental (treatment with tunicamycin or thapsigargin), results in homeostatic alterations in the ER. In turn, this results in increased expression, translation, and secretion of PSP/reg. PSP/reg likely exerts its downstream effects in both an autocrine and paracrine manner. We hypothesize that PSP/reg likely counterbalances the effects of ER stress by simultaneously promoting beta cell proliferation and pro-survival pathways.

ER stress is increasingly recognized as a significant pathologic component of beta cell dysfunction and beta cell death in diabetes^5–8^. Yet there are currently no effective therapies for mitigating beta cell pathology that target the ER. Furthermore, there are no convenient ways to monitor ER health *in*-*vivo*. Wolfram syndrome is a rare, monogenic form of diabetes that stems from beta cell ER dysfunction. As such, animal and cell models of Wolfram syndrome serve as unique tools for identifying biomarkers of ER dysfunction. Our results demonstrate the proteomic alterations that occur in islets undergoing ER stress. More specifically, our data identify PSP/reg as a novel secreted protein that may serve as a biomarker for beta cells experiencing ER dysfunction, as in Wolfram syndrome. We propose that ER stress, whether genetic or environmental, results in homeostatic alterations to the ER that result in increased expression, translation, and secretion of PSP/reg. PSP/reg likely exerts its downstream effects in both an autocrine and a paracrine fashion to counterbalance the effects of ER stress by promoting beta cell proliferation and pro-survival pathways (Fig. 3).

In this study, we applied a proteomic profiling technique involving two-dimensional gel electrophoresis followed by protein identification with mass spectrometry to islets derived from *Wfs1* beta cell-specific knockout mice. This approach allowed us to identify islet-specific proteins upregulated under chronic ER stress through an unbiased approach. Of the differentially expressed proteins identified, special attention was placed on secreted molecules due to their potential to serve as biomarkers of beta cell ER stress.

This proteomic screen identified PSP/reg as a highly upregulated secretory molecule in *Wfs1* knockout islets. PSP/reg is a small (16 kDa) secreted protein that is part of the regenerating protein superfamily and is primarily expressed in the pancreas, and to a lesser extent, in the gastric mucosa and the kidney^32^. It functions as a C-type lectin and thus contains a carbohydrate-binding protein domain that requires calcium for its activity^33^. While the exact function and mechanisms of PSP/reg activity in beta cells remains unclear, previous reports have demonstrated that PSP/reg is abundant in islets regenerating after partial pancreatectomy^22^. Intriguingly, more recent studies of diabetic murine models are also beginning to highlight PSP/reg as a molecule of interest in the pancreas under diabetic states.

Pérez-Vázquez *et al*. recently published a proteomics study of *db*/*db* mouse pancreata, where they found increased expression of PSP/reg, chymotrypsinogen B, and pancreatic alpha-amylase^34^. In this report, PSP/reg was increased by 2.5-fold in *db*/*db* mice. Qiu *et al*. published a similar study examining the pancreata of diet-induced diabetic mice and found PSP/reg to be upregulated by 1.2- to 4.6-fold in diabetic animals^35^. In our proteomic study of Wolfram syndrome, PSP/reg was increased by 3.5- to 4-fold in *WFS1* beta cell specific knockout islets compared to controls. Collectively, these proteomic studies suggest that there are common pathways activated in islets under diabetogenic conditions that converge on increased PSP/reg expression. ER stress is the likely culprit. The fact that PSP/reg is upregulated across three different diabetic mouse models in which ER stress is strongly implicated indicates that PSP/reg may serve as a signal of ER-stressed beta cells in diabetes^36,37^. Indeed, induction and secretion of PSP/reg may be part of a common pathway activated by islet perturbations associated with ER dysfunction.

We hypothesize that PSP/reg is of broader significance to diseases involving ER stress beyond Wolfram syndrome. Accordingly, our data demonstrate that PSP/reg is induced by two mechanistically distinct ER stress-inducing agents (i.e. tunicamycin and thapsigargin). Although this is consistent with our understanding of Wolfram syndrome as a prototype of ER stress disease, the fact that PSP/reg exhibits slower temporal induction dynamics than canonical ER stress genes such as *BiP*, *Chop* and *Txnip* suggests that PSP/reg may function in a different capacity, and towards a different goal, than these other genes. Given that PSP/reg is a secreted molecule, it is possible that its induction at later stages of prolonged ER stress may serve a dual purpose. In addition to signaling ER dysfunction to other cells, beta cell induction and secretion of PSP/reg may also contribute to a cell autonomous adaptive response. Curiously, we found *Reg1* to be induced more robustly by thapsigargin than tunicamycin. This indicates that PSP/reg expression is particularly sensitive to cellular calcium fluctuations, which is consistent with PSP/reg being a C-type lectin with calcium-dependent activity. Further studies are required to determine the targets of PSP/reg’s activity.

Initially, PSP/reg was suggested to be a marker of pancreatic injury and recurrent pancreatitis^38^. Serum PSP/reg is robustly increased in patients with acute or chronic pancreatitis, with some potential for false positives in patients with chronic renal failure under hemodialysis, malignancies of the digestive system or hepatic dysfunction^39^. Elevated levels of PSP/reg have also been recently reported in ventilator-associated pneumonia, chronic obstructive pulmonary disease exacerbation and post-traumatic sepsis^40^. At baseline, however, PSP/reg is mostly secreted by pancreatic acinar cells, while islets appear to only produce PSP/reg under pathologic conditions^17,41^. Bonner *et al*. recently demonstrated that beta cells undergoing apoptosis induce PSP/reg expression in surviving neighboring beta cells, suggesting that PSP/reg signals in a paracrine fashion to promote survival^42^. Additionally, administration of recombinant PSP/reg peptide has been shown to be protective against the development of autoimmune diabetes in non-obese diabetic mouse models^43^. This protective effect may be tied to findings from Okamoto *et al*., which show that PSP/reg is induced by IL-6 and glucocorticoids, thereby inducing beta cell replication through activation of the cell cycle via *Cyclin D1*^44^. Our study extends these findings to a new model of monogenic diabetes and demonstrates that ER stress also leads to increased expression of *PSP*/*reg* in pancreatic beta cells.

Due to these properties, we proposed that PSP/reg could serve as a clinically useful circulating biomarker of beta cell ER stress, in Wolfram syndrome. Unfortunately, there was no significant difference between subjects with Wolfram syndrome and parental or sibling controls. PSP/reg is secreted by both the pancreatic islets and the surrounding acinar tissues. The islets only comprise 1–2% of the pancreatic mass, and receive about 10–15% of the pancreatic blood flow^45^. There is no way to discriminate islet derived PSP/reg from its acinar counterpart. Therefore, detecting PSP/reg elevations in the serum of subjects with diabetes or Wolfram syndrome is challenging. With these important caveats in mind, we identified 3 subjects in our cohort with remarkably elevated levels of PSP/reg. These patients had relatively severe symptoms of Wolfram syndrome. It is possible that serum levels of PSP/reg may rise according to disease severity and that longitudinal levels of PSP/reg may reflect disease progression. When performing univariate analysis on the serum PSP/reg data, we observed that subjects with higher PSP/reg levels tended to have lower fasting C-peptide levels. However, we did not find any clear correlations to the age of onset of diabetes mellitus, optic nerve atrophy, hearing loss, diabetes insipidus, or the Wolfram Unified Rating Scale. This is not altogether unexpected, given the variability in the phenotypic presentation that is typical in Wolfram syndrome. However, in simpler genetic models of *WFS1* loss of function, PSP/reg is clearly upregulated.

Evaluating the correlation between serum PSP/reg and clinical severity in Wolfram syndrome is unfortunately complicated by the rarity of the disease. Given Wolfram syndrome’s prevalence of 1/500,000 people worldwide, it is difficult to obtain a patient sample size sufficiently large enough to assess the relationship between PSP/reg levels and disease progression. This question has, however, been evaluated in other diabetic cohorts with larger affected populations. Recent studies have demonstrated that serum PSP/reg levels are elevated in type 1 and type 2 diabetes, both of which have well-documented ER stress-mediated pathophysiologic components^46,47^. In addition, a study by Bacon *et al*. revealed that PSP/reg levels are elevated in human subjects with HNF1A-MODY, another genetic form of diabetes associated with increased beta cell ER stress^48^. Altogether, these studies suggest that islet injury secondary to beta cell ER stress may lead to elevated serum levels of PSP/reg. Therefore, continued longitudinal measurement of PSP/reg in patients with Wolfram syndrome will help provide clinical correlations and also help determine which patients would benefit most from this this testing.

Our findings provide new evidence that beta cell ER stress alone is sufficient to induce PSP/reg expression in pancreatic beta cells. As PSP/reg is a small secreted peptide, it holds promise as a biomarker of beta cell ER stress in the pathogenesis of both monogenic and common forms of diabetes.

## Materials and Methods

### Animal experiments

The *WFS1* beta cell specific knockout mouse was generated by crossing floxed *WFS1* exon 8 animals with mice expressing Cre recombinase under the control of a rat insulin promoter (RIP2-Cre)^16,49^. 129S6 whole body Wfs1-knockout mice were a kind gift from Dr. Sulev Kõks. This results in a deletion of amino acids 360–890 of the Wfs1 protein^50^. The Institutional Animal Care and Use Committee at Washington University School of Medicine (A-3381-01) approved all animal experiments performed in this study. All methods were performed in accordance with the relevant guidelines and regulations.

### Isolation of mouse islets

Pancreatic islets were isolated from *WFS1* beta cell specific knockout mice and age matched, littermate, Cre-negative, control mice. Mice were anesthetized and pancreata were incubated for 13 min at 37 °C and shaken 30 times. Undigested acinar tissue was removed by using a 70-μm screen and the recovered tissues were washed twice with ice-cold Hanks balanced salt solution followed by centrifugation at 250 × g for 2 min. Islets were handpicked and preincubated in RPMI 1640 medium containing 10% FBS and penicillin streptomycin (Sigma) before experimentation.

### Proteomic analysis

Protein extraction and 2-dimensional differential in-gel electrophoresis (2-DIGE) were performed at Applied Biomics (Hayward, CA), as described^51^. Pancreatic proteins were extracted and concentrations standardized between 3 and 8 mg/mL. Control samples were labeled with Cy2 and *WFS1* knockout samples were labeled with Cy5. Image scans were carried out using Typhoon TRIO and analysis performed using ImageQuant-5.0; in-gel and cross-gel analyses were performed using DeCyder-6.0 to obtain the ratio change of the protein differential expression (GE, Schenectady, NY). Proteins of interest were selected based on a cutoff ratio more than or equal to 1.5 in the *WFS1* knockout samples. Selected spots were identified by Ettan Spot Picker after DeCyder analysis; protein spots were subjected to in-gel trypsin digestion, peptides extraction, and desalting followed by matrix assisted laser desorption/ionization-time of flight analysis to determine their identity. Proteins of interest were identified using database search (Ingenuity Systems, Redwood City, CA; and PubMed).

### Tissue culture

Rat insulinoma cells (INS-1 832/13) were a gift from C. Newgard (Duke University Medical Center, Durham, North Carolina). INS-1 832/13 cells were cultured in RPMI 1640 containing 10% fetal bovine serum (FBS), 1% penicillin and streptomycin, 1% sodium pyruvate and 50 µM β-mercaptoethanol. ER stress induction was achieved through 4- or 8-hour treatments with thapsigargin at 100 nM (Sigma) or tunicamycin at 10 µg/mL (Sigma). DMSO (Sigma) was used as a vehicle control.

### siRNA Transfection

The *Wfs1* gene was silenced with short interfering RNA (siRNA) using the TransIT-X2 dynamic delivery reagent from Mirus Bio LLC according to the manufacturer’s protocol. The siRNA directed against *Wfs1* was predesigned and inventoried by Origene (catalog no. SR504899).

### Quantitative real-time PCR

Total RNA was extracted by RNeasy kits (Qiagen, Venlo, Netherlands). The RNA was used to prepare cDNA using random primers, and reverse-transcribed with a High-Capacity cDNA Reverse Transcription Kit (Thermo Fisher Scientific, Waltham, MA). Quantitative RT-PCR was performed by monitoring in real-time the increase in fluorescence of the SYBR green dye (Bio-Rad Laboratories, Hercules, CA) as described using the Viaa™ 7 Real-Time PCR System (Thermo Fisher Scientific, Waltham, MA)^52,53^. Gene expression was calculated using the ΔΔCt method and data are expressed as fold change ± SEM. To calculate gene expression, Ct values were first normalized to rat 18S rRNA to calculate the ΔCt, then normalized to the appropriate control (e.g. siScr for siRNA experiments, or corresponding DMSO timepoint for ER stress induction experiments) for calculation of the ΔΔCt. Fold change was calculated as 2^−∆∆Ct^. All qPCR reactions were performed in replicates of four. The primers used in this study were: rat Reg1a, 5′-CTGCCAGGATCACTTGTCCA-3′ and 5′-AGCACTGACACCAAGTAGCC-3′; rat Chop, 5′-AGAGTGGTCAGTGCGCAGC-3′ and 5′-CTCATTCTCCTGCTCCTTCTCC-3′; rat BiP, 5′-TGGGTACATTTGATCTGACTGGA-3′ and 5′-CTCAAAGGTGACTTCAATCTGGG-3′; rat Txnip 5′-CAAGTTCGGCTTTGAGCTTC-3′ and 5′-ACGATCGAGAAAAGCCTTCA-3′ rat 18S rRNA, 5′-AGGTTTGTGATGCCCTTAGATGTC-3′ and 5′-CACACGCTGAGCCAGTCAGT-3′.

### Immunohistochemistry

After the mice were sacrificed, whole pancreata were fixed overnight in 10% neutral buffered formalin (Fisher Scientific), serially dehydrated in ethanol, and processed into paraffin block. The paraffin blocks were cut into 5 µm sections. Pancreatic sections were subjected to antigen retrieval in 10 mM sodium citrate (pH 6). The sections were permeabilized in 0.1% Triton X, blocked in 2% BSA and 10% normal serum^54^. Incubation occurred overnight with goat anti-PSP/reg (Gift from Dr. Rolf Graf) and guinea pig anti-insulin (Cell Signaling). The slides were washed with PBS and incubated with CF™ 594 conjugated donkey anti-goat (Sigma-Aldrich) and Alex Fluor 488-conjugated rabbit anti guinea pig (Fisher Scientific) for 1 hour and mounted in prolong gold antifade mounting medium containing DAPI (Thermo Fisher Sicentific). Florescence was observed via fluorescent microscopy (Leica DM4000 B and DFC350 FX).

### PSP/reg enzyme-linked immunosorbent assay

Enzyme linked immunosorbent assay (ELISA) to quantify human PSP/reg was performed utilizing anti-sera from rabbits and guinea pigs that were immunized against recombinant human PSP/reg protein^42,55^. Serum was prepared by centrifugation. IgG was then purified by affinity chromatography on protein A columns. A sandwich ELISA was designed on 96-well plates. Guinea pig antibody was used to coat the bottom of the ELISA plates. Subsequently, the plates were blocked with BSA, and aliquots of serum were incubated for 2 h. After washing, the wells were incubated with rabbit antibody. Then a phosphatase-coupled anti-rabbit IgG was used^56^. A multiplate reader (Dynatech) was used to monitor the reaction of the phosphatase with the substrate. A relative standard curve using recombinant human PSP/reg protein was used to quantify the subject’s PSP/reg levels^57^.

### Clinical information

Clinical data was collected as part the Tracking Neurodegeneration in Early Wolfram Syndrome (TRACK) study at Washington University (ClinicalTrials.gov # NCT02455414). Wolfram syndrome patients were recruited through the Washington University Wolfram Syndrome International Registry and Clinical Study (https://wolframsyndrome.dom.wustl.edu/). All experimental protocols were approved by the Washington University Human Research Protection Office (IRB ID 201107067 and 201104010). All methods were carried out in accordance with relevant guidelines and regulations. Informed consent was obtained from all subjects or, if subjects are under 18, from a parent and/or legal guardian. Natural history of the symptoms of Wolfram syndrome was collected including age, gender, age of onset of diabetes mellitus, optic atrophy, hearing loss, and diabetes insipidus. *WFS1* gene sequencing was also performed^58,59^. Fasting serum samples were drawn to determine glucose and c-peptide. This serum was also used for purposes of biomarker testing. These samples were obtained during the 2014 Wolfram syndrome research clinic. Parents and siblings were used as unaffected controls.

### Statistical analyses

To determine whether any treatment was significantly different from the control, Graphpad Prism was used to conduct two-tailed paired Student’s *t*-tests on the ∆∆Ct values calculated, prior to log transformation. A *p* value less than 0.05 was considered statistically significant. Box and whisker plot for human PSP/reg data was generated utilizing BoxPlotR online software (http://shiny.chemgrid.org/boxplotr/)^60^.

## Supplementary information

Supplementary Information
