# Supplementary material for: Pancreatic stone protein/regenerating protein is a potential biomarker for endoplasmic reticulum stress in beta cells
Source: Sci Rep. 2019 Mar 26;9:5199. doi: 10.1038/s41598-019-41604-4 (PMC6435683; doi:10.1038/s41598-019-41604-4)
Supplement: Supplementary file 1 — Supplementary Information [file 41598_2019_41604_MOESM1_ESM.pdf]

Pancreatic stone protein/regenerating protein is a potential biomarker for endoplasmic reticulum stress in beta cells

Stephen Stone[1,*], Damien Abreu[2,3,*], Jana Mahadevan[2,#], Rie Asada[2,8], Kelly Kries[2], Rolf Graf[4], Bess A. Marshall[1,5], Tamara Hershey[6], and Fumihiko Urano[2,7,¶]

[1]Department of Pediatrics, Washington University School of Medicine, St. Louis, Missouri, United States of America

[2]Department of Medicine, Division of Endocrinology, Metabolism, and Lipid Research, Washington University School of Medicine, St. Louis, Missouri, United States of America

[3]Division of Biology & Biomedical Sciences, Washington University School of Medicine, St. Louis, Missouri, United States of America

[4]Department of Visceral & Transplantation Surgery, University Hospital Zurich, Switzerland

[5]Department of Cell Biology and Physiology, Washington University School of Medicine, St. Louis, MO, United States of America

[6]Departments of Psychiatry, Neurology, and Radiology, Washington University School of Medicine, St. Louis, Missouri, United States of America

[7]Deparment of Pathology and Immunology, Washington University School of Medicine, St. Louis, Missouri, United States of America

[8]Department of Biochemistry, Institute of Biomedical & Health Science, Hiroshima University, Hiroshima 734-8553, Japan

[*]Stephen Stone and Damien Abreu contributed equally to this work.

[#] Current Address: MilliporeSigma (SAFC), St. Louis, Missorui, United States of America

[¶] Correspondence and requests for materials should be addressed to:

Fumihiko Urano, M.D., Ph.D.

Department of Medicine, Washington University School of Medicine, St. Louis, MO 63110, U.S.A.

Phone: (314) 362-8683; Fax: (314) 362-8265

Email: urano@wustl.edu

Supplementary Figures and Tables

Supplementary Figure 1. Loss of *WFS1* Leads to Induction of PSP/reg. (a) Two-dimensional gel electrophoresis was used to resolve the proteomes derived from islets isolated from two 17-week-old WFS1 beta cell specific knockout mice and two age-matched littermate controls. Control (*WFS1+/+*) samples were labeled with Cy2 (green) and *WFS1* knockout (*WFS1-/-*) samples were labeled with Cy5 (red). Of the approximately 450 spots analyzed in the molecular mass range of 5 – 110 kDa, 72 protein spots showed a difference of 1.5-fold or greater between *WFS1* knockout islets and control islets. Spots 1-12 were selected for protein identification via mass spectroscopy (Table 1). (b) Pancreatic stone protein (PSP/reg) was the most upregulated protein identified in the *WFS1* knockout islets. PSP/reg demonstrated 2 isoforms. The higher molecular weight isoform was upregulated 3.98-fold, while the lower molecular weight isoform was upregulated 3.55-fold. (c) Paraffin sections were from both *WFS1+/+* and *WFS1-/-* mice were stained for insulin (green) and mouse PSP/reg (mReg2) (red). These images indicate that PSP/reg co-localizes with insulin the mouse islet. As expected, mReg2 is also abundant within the pancreatic acinar cells.

Supplementary Figure 2. Univariate analysis of PSP/reg clinical data

Serum PSP/reg levels obtained from the subjects at the 2014 Wolfram syndrome research clinic were compared to (a) fasting c-peptide, (b) age on onset of diabetes mellitus, (c) age of onset of optic nerve atrophy, (d) age of onset of hearing loss, and (e) age of onset of diabetes insipidus. Serum PSP/reg levels were also compared to the Wolfram syndrome Unified Rating Scale (WURS) score. (f) Total WURS score. (g) WURS physical component (h) WURS behavioral component.

Spot Number	Protein Name	Fold Change
1	Chymotrypsinogen B	2.73
2	Chymotrypsinogen B	2.86
3	Trypsin 4 precursor	3.01
4	Pancreatic alpha-amylase (N-Terminal Fragment)	-2.02
5	Pancreatic alpha-amylase (N-Terminal Fragment)	2.93
6	Protein Disulfide Isomerase	3.72
7	Trypsinogen 7 precursor	3.02
8	Trypsinogen 7 precursor	2.99
9	Chymotrypsin Like Elastase	2.5
10	Chymotrypsin Like Elastase	3.31
11	Pancreatic Stone Protein (PSP/reg)	3.98
12	Pancreatic Stone Protein (PSP/reg)	3.55

Supplementary Table 1. Proteomic Profiling of *WFS1* Knockout Islets. Spots were selected based on their degree of upregulation in the knockout (*WFS1-/-*) compared to control (*WFS1+/+*) islets. Protein spots were subjected to in-gel trypsin digestion, peptides extraction, and desalting followed by matrix assisted laser desorption/ionization-time of flight analysis to determine their identity. Proteins of interest were identified using database search (Ingenuity Systems, Redwood City, CA; and PubMed). Spot number correlates to the positions seen in Supplementary Figure 1a.

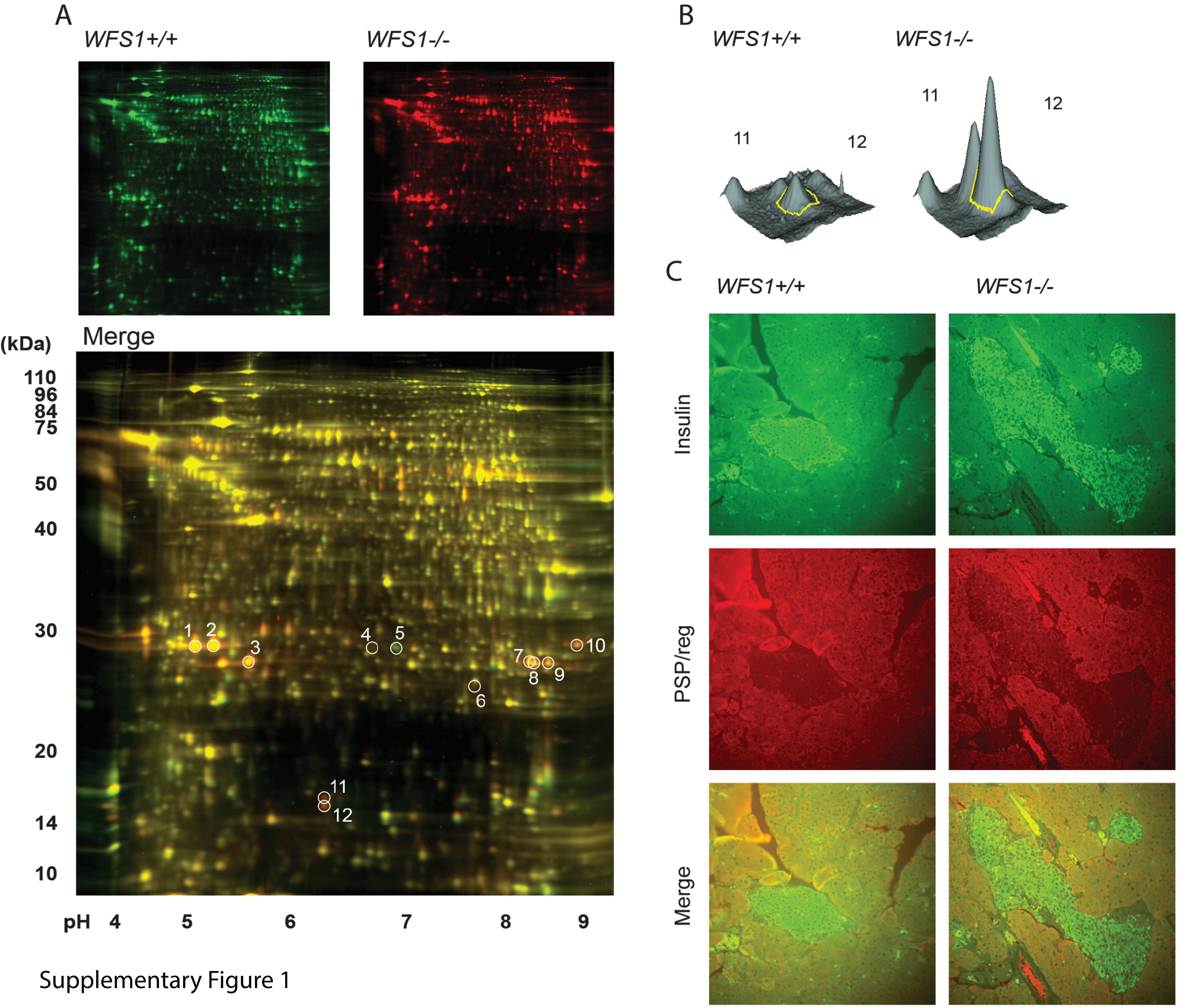

Supplementary Figure 1

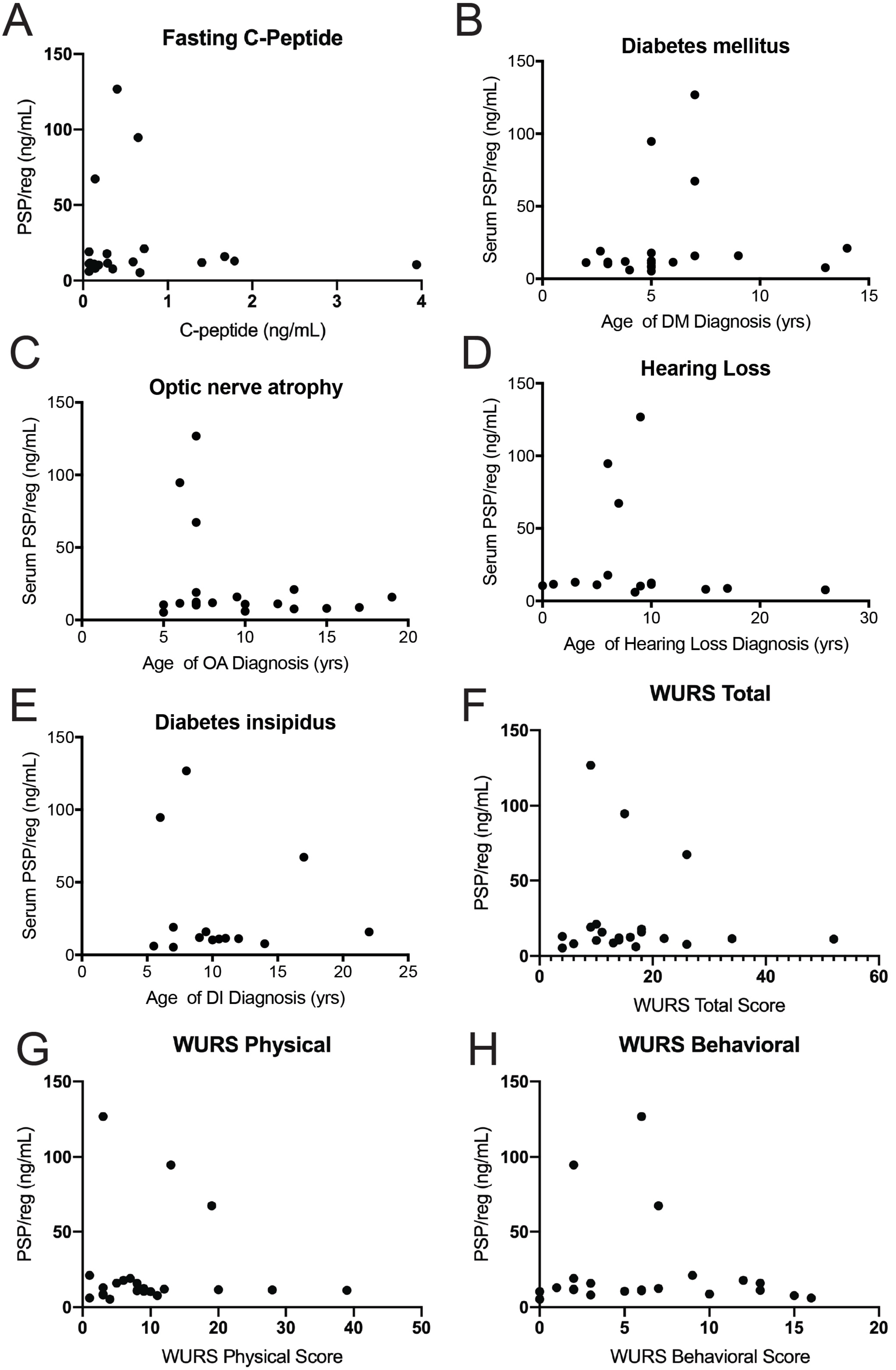

Supplementary Figure 2
